# Cigarette smoking and risk of suicide in bipolar disorder: a systematic review

**DOI:** 10.3389/fpsyt.2023.1179733

**Published:** 2023-05-19

**Authors:** Jesús García-Jiménez, Francisco José Gómez-Sierra, Alicia Martínez-Hortelano, Paula Moreno-Merino, Braulio Girela-Serrano, Patricio Molero, Luis Gutiérrez-Rojas

**Affiliations:** ^1^Department of Psychiatry, San Cecilio University Hospital, Granada, Spain; ^2^Mental Health Unit at La Mata, University Hospital of Torrevieja, Alicante, Spain; ^3^Mental Health Unit at Estepona, Virgen de la Victoria Hospital, Málaga, Spain; ^4^Division of Psychiatry, Department of Brain Sciences, Imperial College London, London, United Kingdom; ^5^Department of Psychiatry and Medical Psychology, University Clinic of Navarra, Pamplona, Spain; ^6^Instituto de Investigación Sanitaria de Navarra, Pamplona, Spain; ^7^Psychiatry and Neuroscience Research Group (CTS-549), Neuroscience Institute, University of Granada, Granada, Spain

**Keywords:** smoking, bipolar disorder, tobacco use disorder, nicotine dependence, suicide, suicidal ideation

## Abstract

**Objective:**

Bipolar disorder (BD) is a highly prevalent, chronic and recurrent mental illness. The smoking rates in patients with BD are much higher than those of the general population, and BD is associated with an increased risk of suicide. An association between smoking and suicidal behavior has been found in the general population, this systematic review examines whether there is evidence of an association between smoking and suicide behavior in patients with BD.

**Method:**

A database search was carried out in Medline, Embase, The Cochrane Library, Scopus, and Web of Science, updated until December 31st, 2021, according to the 2020 PRISMA guidelines. We identified prospective and retrospective studies that included patients diagnosed with BD types I, II, and not otherwise specified, and in which smoking and suicidal behavior were correlated. Articles that focused exclusively on other mental disorders were excluded. The Ottawa-Newcastle scale was used to assess the methodological quality of the included articles.

**Results:**

Fifteen articles (*n* = 7,395) met all the inclusion criteria. In nine of these articles, the authors found an association between smoking and suicidal behavior in BD, while in the remaining six articles, this association was not found. A great deal of variability was observed between articles, particularly in the measurement of suicidal behavior and tobacco consumption. The risk of bias, as assessed by the NOS, was high for most of the included articles, except for two papers, whose risk was low.

**Conclusion:**

It was not possible to establish a clear relationship between tobacco use and the risk of suicide in BD patients due to the heterogeneity of the articles included in this systematic review, which had different sample sizes and methodological issues. However, both conditions are highly prevalent and have a negative impact on the prognosis of BD. Therefore, a systematic approach is needed, based on accurate measurement of a patient’s smoking habits and their risk of suicidal behavior, in order to establish an appropriate therapeutic plan.

**Additional information:**

This research received no specific grant from any funding agency in the public, commercial, or not-for-profit sectors and was registered on PROSPERO with the CRD42022301570 on January 21th 2022.

## 1. Introduction

Bipolar disorder (BD) is characterized by recurrent episodes of depression and hypo/mania alternating with phases of stability or euthymia (1). It has a lifetime prevalence of 2.4% worldwide (2) and is one of the mental disorders with higher rates of disability and worse quality of life (3, 4). BD has a high comorbidity with anxiety disorders and substance abuse (especially alcohol and tobacco) (5) and the risk of suicide is estimated to far exceed that of the general population and patients diagnosed with schizophrenia or unipolar depression (6).

The prevalence of smoking in BD may be as high as three times that of the general population, with up to 70% of BD patients being smokers, compared to 25–30% of the general population (7, 8). A shared genetic vulnerability between smoking and BD has been proposed (9) and some data suggest that nicotine acts on impulsivity and alters key neurotransmitters involved in BD pathophysiology (7, 10). This could explain why nicotine dependence and BD can predict the development of each other (11).

Tobacco use worsens the prognosis of BD patients as smokers have higher cardiovascular and respiratory comorbidity (12), a higher frequency of relapses and hospitalizations (13) and a higher degree of disability compared to non-smokers (14, 15). It is noteworthy that, in contrast to the significant decline in smoking rates observed in the general population, smoking rates among BD patients remain stable and very high, despite the fact that the intention to quit is similar in both groups (16), that smoking cessation improves depressive and anxious symptoms in BD (17) and that it does not seem to increase the risk of relapse (18). The main reason for this situation is that BD patients seem to benefit less from smoking cessation programs, which can be attributed to both system-related factors (fear of decompensation and healthcare professionals who may be poorly motivated to intervene) as well as circumstances related to the disorder (social circles with a high number of smokers and the fact that most patients do not perceive smoking as dangerous) (19).

Suicide is currently among the top 10 causes of mortality worldwide and is a major public health problem as it especially affects young people (15–34 years) (20). Within mental disorders, up to 50% of BD patients will carry out at least one self-harm attempt during their lifetime and 20% will die by suicide, so it is the psychiatric disorder with the highest risk of suicidal behavior (21). The early years of the disorder and depressive relapses are the times of highest risk (22, 23) and it has been noted that suicidal patients have a higher incidence of mixed symptoms, rapid cycling, drop out of treatment more frequently (24) and have a poorer quality of life (25) compared to those who do not make suicide attempts. For all these reasons, addressing suicidality constitutes one of the main current challenges in BD, especially considering that only lithium (26, 27), electroconvulsive therapy (28) and, more recently intranasal esketamine (29), have shown efficacy on suicidal behavior. The relationship between suicidal behavior and BD is complex, and to understand it, a combination of genetic (genes involved in monoamine metabolism), demographic (women make more attempts, but completed suicide is more frequent in men), and clinical factors (such as early-onset BD, depressive polarity, comorbidity, previous attempts, and a family history of suicide) have been proposed (24, 30). Regardless of this, it is always recommended that pharmacological interventions be combined with psychoeducation for both the patient and their family, as well as cognitive-behavioral interventions (24, 31).

A meta-analysis (MA) of prospective studies (32) has found that smoking is associated with a doubled risk of death by suicide in the general population. This risk appears to be even higher in women, which is consistent with other research that has linked smoking with suicide ideation and attempts (33, 34). Certain factors, such as young age, unemployment, the presence of anxious or depressive symptoms, and impulsive personality traits, are common among both patients who smoke and those who make suicide attempts (34). As a result, it has been proposed recently that suicide prevention programs should include the patient’s smoking habit (32). However, there are significant contradictions in the literature analyzing the relationship between tobacco and suicide in BD, with some studies finding positive associations and others finding negative associations (35).

Thus, the aim of this current study is to elucidate further through a systematic review whether there is a relationship between tobacco use and suicidal behavior in BD. For this purpose, the prevalence of smoking and suicidal behavior in the different studies, the methodology used when measuring both phenomena and the existence of possible confounding variables that influence this relationship were analyzed.

## 2. Methods

This study has followed the PRISMA protocol for systematic reviews and meta-analyses and was accepted on the PROSPERO platform on 21-01-2022 with the registration code CRD42022301570. A search was performed using MEDLINE through the OVID tool, Embase, The Cochrane Library, Scopus and Web of Science, with a cut-off date of 12/31/2021. References from systematic reviews and other articles were also reviewed. The search terms were “bipolar disorder OR manic-depressive disorder OR manic depression OR bipolar spectrum” AND “smoke OR smoking OR tobacco OR cigarette OR cigar OR nicotine OR tobacco use OR tobacco use disorder OR electronic cigarette OR vaping OR smokeless” AND “suicide OR suicidality OR suicidal OR self-inflicted death OR completed suicide OR suicide attempt OR suicide ideation OR self-harm.”

The inclusion criteria for this study were as follows: the sample had to consist of patients diagnosed with BD by ICD or DSM (any version), without time restriction, and the measurement of tobacco use and suicidal behavior had to be clearly defined (thoughts of death, ideas of self-harm, attempts or completed acts). Additionally, the association between smoking and suicide had to be described by means of measures of association and their corresponding confidence intervals or *p*-values. Articles that did not analyze BD, those that did not correctly describe the criteria for tobacco use and suicide, and those that analyzed other substances of abuse and self-harm without self-intention were excluded. Systematic reviews, case series, qualitative studies, conference papers, and posters were also rejected.

The articles that initially met the inclusion/exclusion criteria were reviewed through successive stages (Identification, Screening and Eligibility) by two different groups (Group 1 –JGJ and BGS- and Group 2 –FGS and PMM-). Discrepancies were first resolved within each group and then discussed as a whole. If no agreement was reached, the senior researcher LGR was consulted to make the final decision.

The variables of interest were: author, year and country of publication, DSM or ICD diagnosis, sample size, criteria for defining smokers, ex-smokers and non-smokers and their respective prevalence, and definition of suicidal behavior including prevalence (ideation, attempts and completed acts). Also the measures of association between risk of suicide and smoking (OR or RR) or, failing that, statistical value of p.

To measure the methodological quality of the longitudinal studies, it was decided to use the Newcastle-Ottawa Scale (36) that awards a maximum of nine stars to the domains Selection, Comparability, Exposure and Outcomes. This scale was used by each author of Groups 1 and 2 for each article selected. The NOS scale does not allow a proper evaluation of cross-sectional studies, which due to their own design, were considered to provide a lower level of evidence than longitudinal studies.

In order to synthesize the results, we created a table for the articles with a positive association between tobacco and suicide risk and a second table for those works with a negative association. Each table included the following items of interest: Author, country and year of publication, sample size and design, type of DB, definition used to measure tobacco use and suicidal behavior, prevalence of smokers and suicidal behavior and, finally, the strength of association between smoking and suicide (OR or RR).

## 3. Results

### 3.1. Sample size, country of origin of the studies, age of the participants, and type of BD

**Figure 1:**
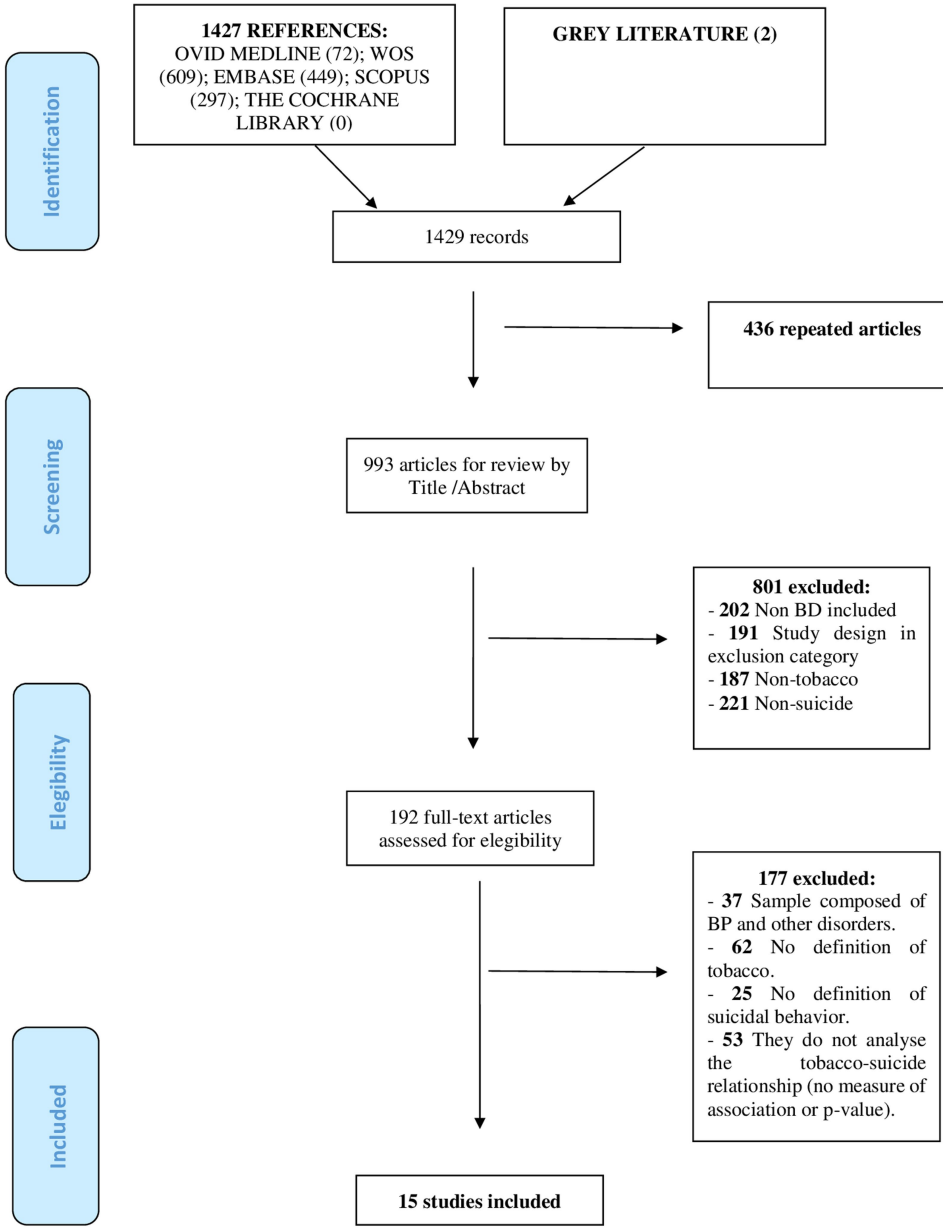
Flow diagram of the systematic review.

The initial search showed a total of 1.429 potential papers which, after applying the corresponding inclusion and exclusion criteria, allowed us to work with a final sample of 15 articles published between 2006 and 2019 (Figure 1). The sample size of the papers ranged from a minimum value of 64 (38) to a maximum of 1,643 (37). Most came from the USA (8), followed by France (2), Israel, Brazil and Italy (1 each) and one multicenter paper from France and Norway was included. All samples were composed of adults except for two papers that included adolescents (39, 40) and the diagnosis of BD was made according to DSM-III-R and DSM-IV criteria (BDI, BDII and BD-NOS).

According to the objectives described in the introduction, some papers that have found a positive tobacco-suicide correlation in BD (41, 42) and others with a negative relationship (13, 43–45) were finally excluded since the methodology did not specify how tobacco use or suicidal behavior was analyzed.

### 3.2. Definition of tobacco use and prevalence

There was a great deal of variety in measuring tobacco consumption and in establishing the categories of active smoker, ex-smoker and non-smoker. The vast majority of authors used the number of cigarettes per day (cig/d) consumed during a given period of time, usually between 1 and 12 months prior to the start of each study. Thus, smokers were those who met both the quantitative criterion (1–100 cig/d on average) and the time criterion, while ex-smokers were those who had consumed that amount but not in the period evaluated and, finally, non-smokers were those who did not meet any criterion.

Only two papers analyzed consumption in terms of nicotinic dependence (46, 47) and a third article distinguished between heavy smokers (≥20 cig/d) and moderate smokers (<20 cig/d) (48). Another publication collected information on alternative forms of consumption such as pipe smoking and snuff (37) and, finally, two publications also analyzed the number of years with maintained tobacco use (48, 49).

The prevalence of smoking (active+ex-smoking) in the different papers ranged from a minimum value of 25% (39) to a maximum of 76.1% (50) (median 46%).

### 3.3. Definition of suicidal behavior and prevalence

Past suicide attempts were the most frequently analyzed dimension of suicidal behavior among the selected papers. One publication did distinguish between active suicidal behavior (self-harm attempts) and passive behavior (death ideation and suicidal ideation) (37) and another one measured the prevalence of suicidal ideation (38). Finally, only one paper analyzed the number of completed suicide attempts (51). The most commonly used source of information was the patient’s clinical history, but specific scales such as the Suicide Ideation/Intent Scale and the Lethality Rating Scale (38), the Suicide Behaviors Questionnaire (52) and the semi-structured interview SCID (49) were also used.

The range of prevalence for suicidal behavior among papers with retrospective design was from 21.3 to 57.8% (median 36.3%). For studies with a prospective design, the duration of follow-up varied, with incidence rates of suicide attempts of 6.9% (52) over 9 months, 14% (40) over 12 months, and 19% (38) over 24 months.

### 3.4. Association between tobacco use and suicidal behavior

A total of 9 publications found a positive association between smoking and suicidal behavior, among which only one had a prospective design (52). Regarding the measures of association, the highest OR value was 5.25 (95% CI: 1.2–23.5; *p* = 0.031) (52) and the lowest of 1.35 (95% CI = 1.05–1.76; *p* = 0.022) (37). One of these papers further indicated that nicotinic dependence was one of the variables with the greatest influence on suicide risk, second only to sex and rapid cycling (47). However, it should be noted that in another work the tobacco-suicide association was only positive for active smokers, but not for ex-smokers (37) and that in another publication this association was only obtained in active smokers with high nicotinic dependence according to the Fagerström test (46). Finally, the initial association between smoking and suicide was maintained in the majority of the original articles after adjusting for age, sex, age of onset, lifetime prevalence of alcohol/substance use, and psychopathological severity among other confounding factors, except in one of these studies whose association was positive in the univariate analysis but not in the multivariate analysis (50) (Table 1).

**Table 1:** Original articles with positive association between tobacco and suicide in bipolar disorder patients.

Author and year	Sample description Country	BD type and design of study	Level of evidence and risk of bias (Newcastle-Ottawa Scale)	Tobacco’s definition	Suicide’s definition	Smoking prevalence and suicidal behavior	Confounding variables	Association measures
Ostacher et al., 2006 (53)	399 adults USA	DSM-IV (BDI, II, NOS and others) Cross-sectional Retrospective	Low level of evidence	Self-report Smoker = At least 1 cig/d currently (daily) or in the past (ever) vs. non-smokers	Past suicide attempts	Smokers (daily or ever) =38.8% Suicidal behavior =33.6%	Age of first depressive/manic episode, past suicide attempts, alcohol/substance dependence lifetime, GAF and CGI score	LROR = 2.25 (95% CI = 1.31–3.86; *p* = 0.003)
Goldstein et al., 2008 (39)	446 adolescents USA	DSM-IV (BDI, II and NOS) Cross-sectional Retrospective	Low level of evidence	Self-report Smoker = At least 1 cig/d currently (daily) or in the past (ever) vs. non-smokers	Past suicide attempts	Smokers (daily o ever) =25% Suicidal behavior = 29.6%	Age, sex, race, intact family	LR OR 3.0 (95% CI 1.7–5.5; *p* = 0.001)
Ostacher et al., 2009 (52)	116 adults USA	DSM-IV (BDI and BDII) Cohorts (smokers vs. non-smokers) Prospective 9 months	Selection: +++ Comparability: + Outcome: +++ Conclusion: Low risk of bias	Self-report Smoker = Any consumption in the month prior to the start of the survey vs. non-smokers	Number of attempts during 9 months of follow-up Suicide Behaviors Questionnaire (SBQ)	Smokers = 27% Suicidal behavior =6.9%	Sex, age of first depressive/manic episode, alcohol/substance dependence lifetime, anxiety disorder lifetime-prevalence, BIS and SBQ score	OR = 5.25 (95% CI: 1.2–23.5; p = 0.031) No association when the variable “impulsivity” was entered into the regression.
Baethge et al., 2009 (51)	352 adults Italy	DSM-IV (BDI and BDII) Cross-sectional Retrospective	Low level of evidence	Self-report Smoker = Daily consumption in the previous six months. The number of cig/d vs. non-smokers	Gestures, attempts or completed suicides (anything but suicidal ideation)	Smokers = 46% Suicidal behavior = 21.3%	Sex, marital status, education level, employment, psychiatric family history, age of onset of disease, diagnostic subtype, alcohol/substance abuse, number of depressive/manic episodes and rate of hospitalization per year	LR OR = 1.89 (95% CI 1.04–3.44;*p* = 0.036)
Baek et al., 2013 (37)	1.643 adults USA (NESARC)	DSM-IV (BDI and BDII) Cross-sectional Retrospective	Low level of evidence	Self-report Smokers = Current (consumption in the previous 12 months), former (no consumption in previous 12 months) and never. Cigarettes, pipe, chewing and snuff.	Active = Suicide attempts during the worst depressive episode. Passive = Thoughts of death or suicidal ideation during the worst depressive episode	Smokers (current or ever) =58.8% Suicide attempts at the worst relapse =22%	Age, sex, race, diagnostic subtype, anxiety disorder lifetime-prevalence, alcohol/substance lifetime-prevalence	Current smoking and history of suicide attempts OR = 1.35 (95% CI = 1.05–1.76; *p* = 0.022) after adjusting for confounding factors. Lifetime smoking (current + ever) no association.
Mathews et al., 2013 (50)	121 adults USA (STEP-BD)	DSM-IV (BDI, BDII, BDNOS) Cross-sectional Retrospective	Low level of evidence	Self-report Smoker = Current (any no. of cig-collected as PPD-in the 2 months prior to study initiation), former (any consumption but not in the previous 2 months) and never (never use).	Past suicide attempts	Smokers (current or former) =76.1% Suicidal behavior =48.8%	Age, ADE and MADRS score, past suicide attempts, alcohol/substance current use	(1) Past suicide attempts were associated with current smoking. OR = 2.27 (95% CI = 1.08–4.76; *p* = 0.030) in univariate analysis but not in multivariate analysis. (2) Suicide attempts were more frequent in current smokers than in former smokers. OR = 2.68(95% CI = 1.15–6.28;p = 0.022)
Ducasse et al., 2015 (46)	453 adults France	DSM-IV BDI and II Cross-sectional Retrospective	Low level of evidence	Self-report Smoker = Current (more than 100 cig/d in the last month, former) (same but not in the last month) and never (exclusion) Nicotinic dependence (severe if Fagerström≥7).	Past suicide attempts	Smokers (current or former) =54.96% Past suicide attempts =39.51%	Sex, education, BMI, thyroid dysfunction, comorbid anxiety disorder, current depression level, bipolar subtype, CTQ score	LR Severe nicotine dependence and history of suicide attempts. OR = 2.80 (95% CI 1.34–5.88;p = 0.02)
Bobo et al., 2018 (47)	1.465 adults USA	DSM-IV BDI and II Cross-sectional Retrospective	Low level of evidence	Self-report Nicotinic dependence according to DSM-IV	Past suicide attempts (≥1 vs. 0)	Nicotinic dependence =39% Suicidal behavior = 32.0%	The authors used a GBM model instead of a conventional logistic regression	LR OR = 1.73 (95% CI 1.38–2.17;*p* < 0.001) Relative influence on the R of suicide = 9.90% (3rd variable with the highest impact, female sex, maximum value = 11.11%).
Icick et al., 2019 (54)	916 adults France and Norway	DSM-IV BDI and BDII Cross-sectional Retrospective	Low level of evidence	Self-report Current or past daily consumption vs. never. In addition, current measured number of years smoking at least one pack/d.	Past suicide attempts (single vs. recurrent)	Current smokers = 50% Suicidal behavior (single and recurring) =37%	Marital status, rate of mood episodes per year, history of mixed episode, SUD groups, 1st-degree family history of mood disorder	LR Tobacco use and recurrent suicide attempts OR = 1.75 (95% CI 1.16–2.63;*p* < 0.01)

ADE, Affective Disorder Evaluation; BD I and II, Bipolar Disorder I and II; BIS, Barratt Impulsiveness Scale; BMI, Body Mass Index; CGI, Clinical Global Impression; CI, Confidence Interval; DSM-IV, Diagnostic Statistical Manual; CTQ, Childhood Trauma Questionnaire; GAF, Global Assessment of Functioning; GBM, Gradient Boosting Machine; LR, Logistic Regression; MADRS, Montgomery Asberg Depression Rating Scale; OR, Odds Ratio; SBQ, Suicide Behaviors Questionnaire; SUD, Substance Use Disorder.

On the other hand, six papers detected no association between tobacco use and suicidal behavior, of which two analyzed the temporal evolution of patients (38, 40). The first one could not relate smoking to suicide risk either at baseline or after 24 months of follow-up (38), whereas in the second paper, adolescent smokers also did not develop an increased incidence of suicidal behavior (40). Another group found no association when dividing the sample into smokers, ex-smokers and non-smokers nor when they performed a dimensional approach according to the number of cig/d consumed in lifetime (49). In the other articles in this section there was also no association between smoking and suicide risk in BD (48, 54, 55). All those data are summarized in Tables 1, 2.

**Table 2:** Articles with no association between tobacco and suicide risk in patients with bipolar disorder.

Author and year	Sample description Country	BD type and design	Level of evidence and risk of bias (Newcastle-Ottawa Scale)	Tobacco’s definition	Suicide’s definition	Smoking prevalence and suicidal behavior	Association measures
Galfalvy et al., 2006 (38)	64 adults USA	DSM-III-R (BDI and II) Cohorts (previous suicide attempts vs. non-previous suicide attempts) Prospective 24 months	Selection: +++ Comparability: + Outcome: ++ Conclusion: Low risk of bias	Self-report Smoker = No. of cigarettes/d in the previous 3 months.	Suicide attempts during 2 years of follow-up. Ideation: Scale for suicide ideation Attempts: Suicide attempt scale and Lethality Rating Scale	Smokers = 45.3%. Suicidal behavior: -Basal = 57.8% previous attempts. -During the 2 years:19% made at least one attempt.	Regression: Tobacco use and suicidal behavior were not associated either at baseline (*p* = 0.46) or during follow-up (*p* = 0.31). No OR
Heffner et al., 2012 (40)	161 patients (80 adolescents and 81 adults) USA	DSM-IV (hospitalized 1st manic episode) Cohorts (smokers vs. non-smokers) Prospective 12 months	Selection: +++ Comparability: + Outcome: ++ Conclusion: High risk of bias	Self-report Smoker = At least 1 pack per day in the previous 30d (PPD). Non-smoker = 0 PPD in the previous month.	Suicide attempts during 12 months of follow-up.	Smoking = 36% adolescents and 56% adults. Suicidal behavior = 10 adolescents (14%) made at least one attempt and only 1 adult.	Regression: Adolescents: OR = 2.02 (95% CI 0.45–9.01; *p* = 0.36) Adults: Could not be calculated
Kreinin et al., 2012 (48)	101 adults Israel	DSM-IV (BDI) Cross-sectional Retrospective	Low level of evidence	Self-report Current smoker = Daily consumption previous 6 months -Heavy smokers:>20cig/d -Moderate: ≤20cig/d Non-smokers = Never smoked or had stopped one month prior to the study. Heavy, moderate or none	Number of attempts in the past	Smoking = 53.9% Suicidal behavior = 36.3%.	No regression *p* = 0.61
Finseth et al., 2012 (55)	206 adults Norway	DSM-IV (BDI and BDII) Cross-sectional Retrospective	Low level of evidence	Self-report Smoker = Daily tobacco use	Number of previous attempts	Smoking = 59.22%. Suicidal behavior = 45.15%.	LR *p* = 0.161
Icick et al., 2017 (56)	616 adults France	DSM-IV (BDI and BDII) Cross-sectional Retrospective	Low level of evidence	Self-report Smoker = Current (at least 5cig/d in the previous 3 months); former or never	Past suicide attempts	Smokers (current or former) = 61.3% Suicide attempts at worst relapse = 36.2%.	LR *p* = 0.078
Medeiros et al., 2018 (49)	336 adults Brazil	DSM-IV (BDI and BDII) Cross-sectional Retrospective	Low level of evidence	Self-report Smoker = Current (>100 cig lifetime and smokes at least 1 cig/d) (CDC definition “current every day smoker”), former or never. In addition, they calculated no. of cig/lifetime by multiplying no. of cig/d by duration of consumption.	Past suicide attempts (*via* semi-structured interview SCID)	Smokers (current or former) = 42.3%. Mean number of cig/d = 20.2 ± 10.7 Suicidal behavior = 41.2%	LR (1) Categorical approach: No difference between current, former and never in terms of previous suicide attempts (*p* = 0.223). Neither between current and non-smokers (former+never) (*p* = 0.093). (2) Dimensional approach: No. of cig/d Spearman correlation coefficient (*p* = 0.133)

BD I and II, Bipolar Disorder I and II; DSM-IV, Diagnostic Statistical Manual; LR, Logistic Regression; OR, Odds Ratio; SCID, Structured Clinical Interview for DSM.

Most of the longitudinal articles were found to have a high risk of bias according to the NOS scale, except for one study in the positive association group (52) and another study in the negative association group (38), which had a low risk of bias. Articles with a cross-sectional design were systematically considered as low level of evidence, as specified in the methods section.

## 4. Discussion

This systematic review analyzed the relationship between tobacco use and suicidal behavior in BD. Of the 15 included papers, nine found a positive association (37, 39, 46, 47, 50–54) whereas six others found no association (38, 40, 48, 49, 55, 56). In addition, a large methodological variability was observed among the studies, that may make it difficult to draw conclusions from this work.

The articles with positive tobacco-suicide association had significantly larger sample sizes (mean = 656.7) compared to those with negative association (mean = 247.3), which decreases the likelihood of random error. However, a greater proportion of hospitalized patients were included in the former, which may introduce selection biases. Suicidal behavior was mostly measured through the number of previous suicide attempts according to the clinical notes, despite the fact that the literature shows that, in many cases, after a self-harm attempt, the patient does not report it or seek emergency services (43). In addition, few studies have included information on death ideation, self-initiated suicide, and completed suicide, i.e., the dimensions that make up suicidal behavior, all of which can result in significant loss of information (57). Currently, there is no gold standard for measuring a patient’s suicide risk (Table 3). Therefore, care consists of taking a complete patient history to detect risk factors, such as previous suicide attempts or lethality, protective factors, such as social and family support or religious convictions, and to analyze whether there is psychiatric comorbidity (BD and major depressive disorder stand out as the pathologies with the highest risk of suicide) (62). Scales can support these interventions, although those currently available have limited predictive value (58). Therefore, scales that analyze all dimensions of suicidal behavior, such as the Columbia scale (63), are recommended. In short, as there are currently no tools available to clearly differentiate between a patient who is going to make a suicide attempt and one who is not (57), the approach should be based on gathering as much information as possible.

**Table 3:** Assessment and treatment of suicidal behavior in bipolar disorder patients: based on Schreiber and Culpepper, 2021 (58).

Risk factors	Protective factors
Previous attempts Psychiatric comorbidity Hopelessness Marital status separated, single, widowed Unemployment Professions: Nursing and medical Physical comorbidity Pain Neurological disorders Childhood abuse Family history	Social support Family support Pregnancy and parenthood Religious convictions
Use of scales	
SAFE-T (Substance Abuse and Mental Health Administration, SAMHSA)	5 steps, assesses risk factors, protective factors, asks about ideation and plans.
Columbia Suicide Severity Rating Scale(C-SSRS) (57, 63)	Dichotomous scale, evaluates intensity of suicidal ideation and self-injurious behavior.
Treatment	
Lithium (26)	Effective but frequent side effects and risk of poisoning
Antidepressants (59)	Frequent use but limited effectiveness in acute phase
Esketamine (29)	Hospital administration, little data on actual clinical practice
ECT (60)	Requires hospital admission, quickly effective
Cognitive behavioral therapy (61)	Reduces the probability of a new autolytic attempt

Regarding tobacco use, each paper used different criteria based on the number of cigarettes and duration of consumption, with the exception of one that adhered to the standardized criteria of the Centers for Disease Control and Prevention (49, 64). Three authors measured the degree of nicotine dependence (46–48), while only one included information on alternative forms of tobacco consumption such as pipe smoking, snuff use, or chewing tobacco (39). The variability in measurement criteria between the different articles makes it difficult to make comparisons. Developing standardized recommendations for future studies (Table 4) may be advisable. For instance, future studies could quantify the number of daily cigarettes and total duration of tobacco consumption to establish a general classification (64), or use the Packs/year index (PPI) (65) to assess the risk of developing tobacco-related diseases and the probability of quitting smoking. Although these parameters are relatively simple to obtain, they have limitations in establishing differences between smokers in terms of the actual amount of tobacco consumed. It is known that both the number and intensity of puffs can differ between smokers (70). Therefore, it may be preferable to use laboratory techniques such as cooximetry (71) or determining the levels of cotinine in blood and other fluids. Cotinine is a nicotine metabolite with a longer elimination half-life, and its levels can provide more accurate information about the actual amount of tobacco consumed (72). As outlined in (Table 4), these techniques allow for the objective differentiation between smokers, ex-smokers, and passive smokers, although they have certain drawbacks such as economic costs and limitations inherent in the techniques themselves. Additionally, it is possible to determine the patient’s degree of nicotine dependence, both physically with the Fagerström test (68) and psychosocially with the Glover-Nilson scale (69). Both tests are cost-effective and diagnostically valid options as they help to determine the probability of success of interventions aimed at helping the patient to quit smoking. Also, in the original articles, there was also a great variability in the time of exposure to tobacco. Two types of approaches were observed: a cross-sectional approach in which only current consumption was asked (39, 53–55), and a longitudinal approach that collected the history of consumption over varying time periods, such as one month (40, 46, 52), two months (50), three months (38, 56), six months (48, 51), twelve months (37), and lifetime smoking rate (49). It is logical to point out that the longitudinal criterion is better at discriminating the authentic smoker, and the longer the observation period selected, the better it does so.

**Table 4:** Instruments to measure tobacco consumption: advantages and disadvantages.

Current CDC criteria for smoking population (64)
Never Smokers – Adults who have never smoked a cigarette or who have smoked fewer than 100 cigarettes in their entire lifetime. Former Smokers – Adults who have smoked at least 100 cigarettes in their lifetime, but say they currently do not smoke. Nonsmokers – Adults who currently do not smoke cigarettes, including both former smokers and never smokers. Current Smokers – Adults who have smoked 100 cigarettes in their lifetime and currently smoke cigarettes every day (daily) or some days (non-daily).

Instrument	Values	Interpretation	Advantages	Inconveniences
Index of packages/year (IPA) (65)	[(No. of cig/d x no. of years)/20 (number of cigarettes in a pack)]	The higher the value, the higher the risk of pathologies and the lower the probability of abandonment.	Easy to measure.	Does not measure the particularities of each smoker
Cooximetry (66)	Smokers: ≥10 ppm of CO in exhaled air. Sporadic smokers: 6–10 ppm CO Non-smokers: <6 ppm CO	Indicates type of smoker. Probability of tobacco-related pathology.	Objective Simple technique Low cost	Requires instrumentation Can be altered by environmental conditions
Cotinine (67)	Saliva:10-25 ng/mL Serum:10-20 ng/mL Urine:50-200 ng/mL	Indicates type of smoker and probability of pathology.	Objective Does not depend on the patient’s voluntariness (patients with pulmonary pathology).	Requires laboratory techniques (inexpensive) Its values have been modifying over time
Degree of dependence				
Physics (Fagerström) (68)		6 items = Quantity of cigarettes, compulsion and dependence. Value 0–10, the higher the value, the higher the dependence. Simple instrument that indicates the patient’s physical dependence and predicts the probability of quitting smoking.		
Psychological (Glover-Nilsson) (69)		11 items = Assesses psychological and behavioral changes in the patient associated with tobacco use. <12 Mild; 12–22 Moderate; 23–33 Strong; >33 Very strong.		

CDC, Centers for Disease Control and Prevention.

The results of this study suggest that the prevalence of smoking in BD (25–76%) and suicidal risk (21–58%) is high, which is consistent with a recent meta-analysis in the general population indicating that smoking may increase the risk of suicide by up to two times, particularly among women (32). However, neither the etiology nor the causality of this relationship has yet been clarified, so different explanations have been proposed. First of all, smoking and suicidal behavior share common genetic pathways (9) and lower serotonin levels have been found in the hippocampus of smokers (73). In addition, nicotine could increase the risk of suicide because it alters the function of MAO (74), a key enzyme in the metabolism of monoamines, and because it produces a continuous and inappropriate activation of the hypothalamic–pituitary–adrenal axis (75). On the second place, an inflammatory pattern similar to that found in mental disorders predisposing to suicide, such as schizophrenia or unipolar depression, has been described in smokers (76). This pattern involves an increase in proinflammatory products such as IL-6 and TNF-α (77), as well as a reduction in antioxidant enzymes such as paraoxonase (78). Comorbidity is another important factor when analyzing the tobacco-suicide relationship. Thirdly, tobacco smoke-induced tissue hypoxia predisposes individuals to physical cardiovascular and pulmonary diseases, which can contribute to a reduced quality of life (79), and then smokers have higher rates of severe mental disorders and more impulsive personality traits, two elements that also predispose to suicide risk (80, 81). In fact, the initial association between smoking and suicide risk that had been found by two of the papers included in this review did not remain significant after adjusting for impulsivity traits (52), substance abuse, and depressive symptoms (50). Finally, some authors have proposed that nicotine would help to combat the cognitive impairment experienced by some patients and the sedation produced by pharmacological treatments by granting a therapeutic function to tobacco use (82).

In fact, multivariate analysis shows that the relationship between tobacco use and suicide risk can be influenced by the presence of sociodemographic and clinical variables. Among the first group are the sex and age of the patient, as well as the age of onset of the disorder, while the history of alcohol/substance use, anxiety disorders and the diagnostic subtype of BD were the most repeated clinical covariates in the original articles (Table 1). It should be noted that only three of the papers included in the review (37, 51, 52) included most of these covariates, so it is important that future papers consider this complex tobacco-suicide relationship in their analysis.

One of the objectives of highlighting the high prevalence of suicide and smoking in BD is to propose a series of interventions that can help reduce their impact. Regarding tobacco use, data indicate quitting smoking is associated with better physical and emotional well-being and does not increase the risk of relapse, both in the general population and in patients with mental disorders (83). The approach should be done systematically, combining pharmacological and psychotherapeutic techniques, and informing the patient that this is a long process in which relapses may occur (84, 85). First, it is important that the patient is clinically stable and that no major life changes or treatment changes are expected in the short term (86). Next, the current consumption should be recorded, preferably using the Fagerström test and the number of previous quit attempts, as they help to predict the need for more intensive interventions (87). A successful intervention is one based on the 5 A’s: “ask,” “advise,” “assess,” “assist,” and “arrange” (88). It has been found to be equally valid to set a quit day as to progressively reduce tobacco use (72, 89). Clinical guidelines recommend that pharmacological treatment should always be offered to BD patients, even in the case of mild dependence (86), and that the option with the most evidence is varenicline (18, 90) followed by nicotine substitutes and bupropion (91). Other strategies include measuring the pulmonary damage to make BD patients aware of the harm that smoking causes to their health (92, 93).

In any case, the patient’s treatment should be reviewed for possible interactions and combined with psychosocial support through emotional interviewing, psychoeducation and cognitive behavioral techniques, all with similar efficacy (94). Finally, adequate follow-up should be scheduled, especially at baseline when the risk of relapse is maximal (95), but also in the long term to assess changes in psychopathology, metabolic profile (96) and to adjust the dosage of certain drugs such as clozapine (97).

### 4.1. Limitations

This study has a number of limitations that should be pointed out. Firstly, the articles included in this review are primarily from the USA and Europe, and thus, it does not analyze the possible tobacco-suicide relationship in BD patients from other geographic areas. This may have influenced the results, as the prevalence of smoking in samples from Asia is lower than that of Western patients (32.4% vs. 52.4%, respectively) (98). Next, this review also does not compare with other mental disorders in which suicide and smoking are also common, such as depression or schizophrenia. Although this question was initially raised, it was decided not to include it to facilitate the applicability of the results. Additionally, some papers that have found a positive tobacco-suicide correlation in BD (41, 42) and others with a negative relationship (13, 43–45) were finally excluded since the methodology did not specify how tobacco use or suicidal behavior was analyzed. Finally, no papers analyzing the impact of e-cigarette use on BD patients have been found, despite a recent paper pointing out that in the general population, this type of device may increase the risk of suicide in adolescents and young adults, especially in women (99).

### 4.2. Conclusion

This work has highlighted that tobacco use and suicidal behavior are common issues among patients diagnosed with BD and that specific interventions should be implemented to address these problems due to their negative impact on prognosis. Although the literature suggests a relationship between tobacco use and suicide, it is not clear that the smoking habit is the cause of suicide attempts possibly due to significant methodological differences observed among studies. Smoking is probably associated with other variables (biological, clinical symptomatology or personality) that may explain the positive statistical significance with suicide behavior. Instead, this work has analyzed the various ways of measuring both variables with the aim of facilitating future comparisons based on stable and standardized criteria.

## Implications

Suicide is the most common cause of premature mortality in Bipolar Disorder (BD), doubling or tripling those observed among the general population. The association between smoking and the risk of suicidal behavior was also found in several studies. The association between tobacco use and increased suicidal risk in BD may be the result of a complex interrelationship of factors that predispose independently for both conditions. It is not possible to establish a clear relationship between smoking and suicidal behavior. A common methodology based on more objective criteria is necessary to elucidate whether tobacco use is associated with the risk of suicide in patients with BD.

## Author contributions

JG-J, FG-S, LG-R, and AM-H designed the research study. JG-J, FG-S, and AM-H performed the research. PM and PM-M contributed analytic tools. JG-J, FG-S, LG-R, and BG-S analyzed the data and wrote the manuscript. All authors have read and approve the final manuscript.

## Conflict of interest

The authors declare that the research was conducted in the absence of any commercial or financial relationships that could be construed as a potential conflict of interest.

## Publisher’s note

All claims expressed in this article are solely those of the authors and do not necessarily represent those of their affiliated organizations, or those of the publisher, the editors and the reviewers. Any product that may be evaluated in this article, or claim that may be made by its manufacturer, is not guaranteed or endorsed by the publisher.
